# The decision maker’s lament: If I only had some science!

**DOI:** 10.1007/s13280-024-01986-w

**Published:** 2024-03-11

**Authors:** Gustavo A. Bisbal

**Affiliations:** United States Department of the Interior, United States Geological Survey, 917 National Center Room 3A400, 12201 Sunrise Valley Drive, Reston, VA 20192 USA

**Keywords:** Actionable science, Decision making, Environmental management, Usable knowledge

## Abstract

Environmental decision makers lament instances in which the lack of actionable science limits confident decision-making. Their reaction when the needed scientific information is of poor quality, uninformative, unintelligible, or altogether absent is often to criticize scientists, their work, or science in general. The considerations offered here encourage decision makers to explore alternative approaches to alleviate their disappointment. Ironically, many researchers lament the lack of support for the science they wish to deliver and accuse decision makers of failing to realize the value of the scientific studies they propose. Both communities would benefit by remembering that producing actionable science for a pending decision requires knowing the context for that decision beforehand. They may also look inward. Only then will they find answers to the question: What can I do within my own capacity to ensure that the necessary actionable science becomes available and facilitate its use to inform decisions?

## Introduction

Making informed and justifiable decisions is the most coveted goal that decision makers (this term will be used to collectively refer to public authorities, designated officials, agency administrators, and resource managers) responsible for addressing environmental concerns and managing natural resources strive to achieve. But the task is complex and risky. Their decision-making process is often influenced by an intricate assortment of factors including institutional jurisdiction and values, bureaucratic constraints, socio-political circumstances, budgetary realities, market pressures, and public demands among others (e.g., Morrison-Saunders and Bailey 2003; Kiker et al. 2005). These influences leave decision makers in an uncertain state where options may be constrained and offer little confidence in achieving the results they seek to satisfy priority goals within their environmental portfolios. This scenario is far from optimal because an unwise decision could render unacceptable consequences to those who make the decision as well as those affected by it.

Fortunately, many decision makers welcome the contributions that scientific information, tools, and services offer to help them mitigate risk, uncertainty, and speculation (Morrison-Saunders and Bailey 2003; White et al. 2019; Thomas-Walters et al. 2021; Cooke et al. 2023). Their use of science to improve decision making is, in fact, consistent with the intended central role of science contemplated within the field of decision analysis (e.g., Murphy and Weiland 2014; Baker et al. 2022; Hemming et al. 2022). Moreover, in the United States and many other countries, the legal, administrative, and institutional standard for the application of science to decision making instructs regulatory natural resource agencies to include the best available science in the formulation of public policies and planning directives (Bisbal 2002; Sullivan et al. 2006; Ryder et al. 2010; Charnley et al. 2017). Scientific information that not only provides practical value to inform a decision, but is explicitly motivated by the context of that decision, has been generically labeled “actionable science”; a concept abundantly examined in the professional literature (Palmer 2012; Beier et al. 2017; Bisbal 2019; Gerber et al. 2020; Mach et al. 2020).

The appetite for science as critical input to gain decision-making advantage, however, may end bitterly when the anticipated scientific information is of poor quality, uninformative, anecdotal, contradictory, obscurely communicated, or altogether absent. This misalignment between the prospect of valuable scientific knowledge to help better understand the contours of uncertainty and a set of competing decision alternatives can be disempowering and lead to disappointment (Choi et al. 2005). The frustration felt by decision makers when their expectations for science to help reduce both uncertainty and the chances of undesirable outcomes go unfulfilled, can be captured in the form of a generic lament: “If I only had some science!” This sentiment reveals that to many decision makers, the perceived lack of actionable science limits their confidence in decision-making.

Overtly or not, the behavioral response of decision makers to a deficit in actionable science is often to blame scientists, their work, or science in general for their failure to inform and support the decision-making process. While criticisms of this kind are not always shared openly by decision makers, scholars dedicated to characterizing the science-policy divide have documented them through an abundant body of literature, and linked these sentiments to differences in epistemic backgrounds, working philosophies and ideologies, and existing interpersonal emotions and prejudice between scientists and decision makers (e.g., Sarewitz 2004; Choi et al. 2005; Cook et al. 2013; van Stigt et al. 2015; Roux et al. 2006; Dunn and Laing 2017). In such instances, the sense of bewilderment voiced by decision makers is similar to that expressed by applied researchers who are disappointed when proposal reviewers, award adjudicators, and potential science end users show little enthusiasm and dismiss research proposals that, in the researchers’ view, could make a significant contribution toward the formulation of environmental management decisions or natural resource policy action (Bisbal 2022). In both cases, the initial tendency among these two communities—scientists and decision makers—is to blame each other as the real culprit interfering with the accomplishment of their respective objectives (e.g., Choi et al. 2005; Roux et al. 2006). Bisbal (2022) focused on those lamenting researchers and offered steps to address their own shortcomings to providing actionable science. The present contribution is a companion perspective and extends a similar invitation—this time to lamenting decision makers—to consider concrete actions they can take to secure the science needed for confident decision-making.

## Tips to avoid the decision maker’s lament

Decision makers willing to integrate scientific evidence to improve or strengthen the decision-making process understand that their success depends on playing an active role in engaging the knowledge communities they wish to interact with (Raymond et al. 2010). They realize that assuming the posture of detached bystanders and leaving the desired integration of science they can use into their pending decisions entirely to chance is a recipe for disappointment. Instead of lingering in lament when the scientific evidence is insufficient to assist in the evaluation of competing decision alternatives, when the data are scant and inconclusive, or when there are gaps in knowledge, they double down on their commitment toward remedying those failings. Here are a few key considerations and practices that may give decision makers a strategic advantage toward a successful integration of science into decisions:

### Be clear about the decision context

Finding adequate science to inform a decision requires an unescapable first step: to frame the context of the decision of interest (Runge et al. 2020). A pending decision reveals an unsettled action point concerning, for example, the formulation of policy, preparation of a resource management plan, adoption of regulatory legislation, or implementation of strategic interventions. Decision makers are responsible for articulating the contexts for decisions that can then motivate or inspire the scientific activities proposed to address them. This intentional alignment of the scientific contribution tailored to match the problem context as articulated by the decision maker is the cornerstone of actionable science (Beier et al. 2017; Bisbal 2019; Gerber et al. 2020; Mach et al. 2020).

In some cases, decision makers deviate from their central responsibility. Instead of framing the decision context that produces the *demand* for actionable science, they instinctively focus on how to fund the wholesale *supply* of science studies, even if not necessary to inform their decision (McNie 2007). The relationship between available scientific information (supply) and decision context (demand) has been thoroughly acknowledged elsewhere (McNie 2007; Sarewitz and Pielke 2007; Bisbal 2019). Examples of a mismatch between supply and demand are well documented, as illustrated by the weak performance that the Large-Scale Biosphere–Atmosphere Experiment in Amazonia, Brazil, has had in the area of supplying knowledge directly relevant to societal and environmental concerns at the local level in the Amazon region (Lahsen and Nobre 2007). For some decision makers, diverting attention to the attributes of different research methodologies or specialized instrumentation may provide them with a convenient way out of having to frame the decision problem and needing to elaborate on difficult—and often-times sensitive or controversial—aspects of decision making (Bisbal and Eaton 2023). According to Johnson et al. (2015) this behavior may not be a choice but, instead, the product of insufficient literacy in the basic principles and tools of decision science. Regardless of the reason, the departure from the basic duties expected of decision makers leads to a distraction, at best, or a glaring void that prevents the planning and initiation of actionable science projects, at worst. Building capacity to confidently frame decisions is predicated on securing the appropriate mix of decision science competencies through formal education and practical training (Johnson et al. 2015; Hemming et al. 2022).

Savvy decision makers realize that carefully framing the decision context is essential for four reasons: first, it helps signal to various groups or individuals affected by the decision and –importantly– science producers who may wish to assist in the delivery of actionable products, whether the integration of scientific evidence into the decision-making process is anticipated or not. Second, contextualizing the decision also helps refine the disciplinary perspectives and categories of knowledge (Raymond et al. 2010) that may provide the most useful evidence to inform the decision at hand (Cooke et al. 2023). Third, it highlights key uncertainties and critical variables in need of quantification (Runge et al. 2020), and offers valuable details to recognize configurations of the decision space such as time frames, geographic scale, and governance scope (Baker et al. 2022). The fourth reason reveals important legal, economic, political, cultural, social, historic, and even religious and moral dimensions that have crucial influence over the implementation of decision options. The integration of all these elements is complicated but indispensable at the same time. Only then can a meaningful conversation start about if and what specific scientific contributions might help inform decisions concerning the environment and natural resources.

### Lead resolutely but listen attentively

The authority to articulate the context of priority decisions falls squarely on the shoulders of decision makers. While this function is the centerpiece of their job description, other individuals or groups who are not legitimate decision makers may find it attractive and will take over the podium if it appears vacant (Bisbal and Eaton 2023). McConnell and ’t Hart (2019) evaluated cases of inaction in public policy and offered a typology of “silences” during policy interventions. Regardless of whether instances of silence among decision makers are the result of dysfunctional flaws or products of calculated strategies, many actors (e.g., scientists, media outlets, industry representatives, activists, non-governmental organizations, interest groups) may capitalize on the opportunity to impersonate seemingly absent decision makers and reshuffle environmental priorities according to their own ideologies and perspectives. These interventions give them control over the science agenda and a louder voice in the process of assigning and mobilizing available resources (Bisbal and Eaton 2023). Many studies document cases in which illegitimate decision makers have gained undue influence in determining public policy priorities and, ultimately, controlling what science to champion or block. The manipulation of scientific evidence by major industries (tobacco, chemical, and pharmaceutical) in support of policies that maximize corporate profits, for instance, helps illustrate this point (Legg et al. 2021). In another example, Lucas (2021) chronicles the pervasive influence that fossil fuel corporations and resource extraction industries have had over climate and energy policy and decision making by the Federal Government in Australia since the 1980s. Decision makers who lament the disconnect between science products and the decisions at their doorstep may reflect on whether their silence may have been seized upon and exploited by others.

Conversely, decision makers committed to clearly articulating the priority decision contexts at-hand will be better positioned to deter illegitimate actors attempting to hijack science planning, the allocation of research funding, and the formulation of decisions. They understand that actively inviting and engaging a diversity of perspectives in the decision-making process is the cornerstone of a deliberative and inclusive democratic co-management arrangement (e.g., Zachirsson 2010). By capturing distinct values, interests, experiences, and ideologies pertaining to different segments of society, scholars have reported important benefits to the definition of the decision context, including enhanced communication, mutual respect, trust, equality, transparency, and pluralism (e.g., Gerber et al. 2020; Zaman et al. 2020; Gluckman et al. 2021). The success of this participatory approach in improving sustainability and conservation programs has been documented in a variety of scenarios around the world (e.g., van Putten et al. 2022; Hamelin et al. 2023; van de Water et al. 2023). Reaching a collective vision for shared natural resource management and the priority decisions ahead, also sets the stage for integrating a broad array of epistemologies and knowledge contributions, including Indigenous knowledge (Wheeler and Root-Bernstein 2020), local knowledge (Raymond et al. 2010; Hamelin et al. 2023), and collaborative disciplinary perspectives across natural and social sciences boundaries (Stock and Burton 2011). Assembled together, they provide a robust body of available evidence to inform priority decisions (Cooke et al. 2023).

### Understand the “best available science” mandate

In many situations, decision makers are expected to use the best available science in their formulation of policy, administrative rulemaking, and implementation of decisions (Sullivan et al. 2006; Ryder et al. 2010). This concept is commonplace in numerous environmental statutes and regulatory precepts, but typically lacks a clear definition of its properties, standards, or practical application in decision making (Bisbal 2002; Charnley et al. 2017). What will the judge rule if the best available science used to inform a decision was challenged in court? Will the public be able to understand, and support, the best available science that favored one decision alternative over another? For years now, scholars have entertained different perspectives and interpretations of what might be considered best available science by dissecting and unpacking the individual component elements within this concept (Bisbal 2002; Sullivan et al. 2006; Ryder et al. 2010; Charnley et al. 2017; Esch et al. 2018). What is deemed *best* (science) and who holds the responsibility to issue a conclusive verdict on that determination? Considering contemporary and past information, or the variety of publication platforms and geographic sources, is there a time period, location of origin, or preferred outlet that help us determine what (science) is *available*? What will be considered and accepted as legitimate *science* in light of an enormous and ever-expanding body of evidence made available to decision makers? Is the integrative nature of interdisciplinary sources of information (Stock and Burton 2011) preferred over the isolated disciplinary domains of more traditional categories of physical, biological, and social sciences? And how are Indigenous and local knowledges factored in (Raymond et al. 2010; Wheeler and Root-Bernstein 2020)? Understanding the many facets surrounding the mandate to use the best available science in a specific decision context helps refine and align the “supply” of evidence (McNie 2007; Sarewitz and Pielke 2007; Bisbal 2019) to the “demand” initially identified by decision makers. This function requires skilled professional assistance as described in the following section.

### Secure and trust a reliable science provider

A primary objective of advancing actionable science is to encourage a fertile interaction between scientists and decision makers so that scientific information can be made available to improve resource management decisions. It is possible that urgency, inexperience, overconfidence, former education, etc., may entice some decision makers to attempt to fully control the process of gathering, analyzing, summarizing, and understanding scientific evidence to inform their own decision response. While the apparent advantages of independence, self-reliance, and expediency may seem attractive, the potential downside can have dire consequences, including poor information, sub-standard interpretation, limited options, and disastrous decisions. These decision makers fail to understand that the delivery of scientific findings to inform decision making cannot materialize effectively unless scientific literacy in human–environment systems and the ability to distinguish sound science from unsound science (or “antiscience”, see Apitz et al. 2017) are strong. Ironically, decision makers who are reluctant to acknowledge their own scientific limitations, intentionally circumvent scientists, or give perfunctory attention to the interface between scientific knowledge and decision-making may, in the end, be responsible for their own information deficiencies.

Prudent decision makers, on the other hand, understand that engaging with scientists is indispensable to realize the benefits of actionable science. From their point of view, securing the advice of a trustworthy, robust, and objective scientific interlocutor is essential. The modality and degree of this engagement vary according to each particular circumstance and span a continuous spectrum that ranges from a limited partnership to a more immersed co-equal participation (Bamzai-Dodson et al. 2021). Several institutional constructs have emerged in the United States and other countries to facilitate the dialogue and effective flow of information between science producers and end users (Gluckman et al. 2021). The nomenclature describing these arrangements introduces “knowledge brokers”, “intermediaries”, “boundary spanners”, “knowledge translators”, or “evidence bridges” (Choi et al. 2005; Cook et al. 2013; Duncan et al. 2020; Gluckman et al. 2021; Kadykalo et al. 2021; Neal et al. 2022). The configuration and nuanced functions of these boundary individuals and organizations have been extensively described in the specialized literature dedicated to the health care, disaster management, education, agriculture, and environmental sectors (e.g., Neal et al. 2022). Their shared objective is to engage with scholars and practitioners alike to identify and address their respective needs, interests, concerns, and perspectives. By bridging these two domains, these intermediaries are best positioned to implement mechanisms for two-way communication, foster trust, and promote more frequent and productive cooperation. As neutral intermediaries, they conduct policy analyses, translate jargon, interpret technical content, and summarize and deliver existing science (e.g., Posner and Cvitanovic 2019).

While outside agents play an important role in the interpretation and use of scientific knowledge during decision making, retaining in-house support from an individual or entity with the necessary professional stature, adherence to ethical and integrity values, and the ability to interpret and summarize scientific outputs could offer an additional layer of scientific advice (Sutherland and Burgman 2015). This approach need not exclude the contributions of boundary arrangements as they can still provide complementary value. Securing a trusted science advisor can offer additional value, such as enhancing the day-to-day discussion of scientific matters or gaining in-house training on the many dimensions of the scientific process. The profile of a science advisor may be quite heterogeneous regarding professional background, level of expertise, and approach to an evidence-based culture. Consequently, a focused recruitment must be tailored carefully to weigh up the pros and cons of specific selection criteria and competencies of relevance to the hiring party. On occasion, the establishment of a scientific advisory board that combines the contributions of qualified experts from academia, industry sectors, government agencies, Indigenous groups, civil society, and other pertinent epistemic communities, may be recommended to gain diversity and a pluralistic view on the weight of scientific evidence. As they seek to engage in a reliable relationship that secures the precepts of good science, savvy decision makers steer clear of scientists who are obsequious or have hidden agendas. Being counseled by scientists who conveniently promote biased information in support of (or against) a specific decision as a way to please their employer or unleash their own ideological beliefs, could have devastating consequences for the decision maker, the scientists involved, and society at large.

### Take a peek into the future

When decision makers are presented with the notion of some scientific output that could improve or strengthen the decision-making process, it may be helpful for them to mentally look ahead and ask in advance what exactly will they receive when the anticipated products or findings become available (Sutherland et al. 2013). At a minimum, they could ask: Will I understand what this scientific information means and the relevance it has in the context of my unresolved decision? Will the product/s be timely to inform the decision? Will I be aware of all the assumptions underlying the work and the constraints they impose on the applicability of the results? Can the results be extrapolated to offer reliable conjectures of value to my specific decision context? Will I know the extent of the uncertainty associated with the results and how it affects the ability to manage risk? Clearly, answering many of these questions requires the involvement of a trusted scientific voice as suggested above.

As the image of the anticipated actionable science output begins to gain clearer definition and meaning, the decision maker will be better positioned to ascertain the intended application of that contribution in the process of selecting the preferred alternative (i.e., formulating the decision to be implemented). After securing a more realistic notion of what is ahead, it is now time to return to the present and determine what scientific efforts to support, whether it is necessary to mobilize resources to prompt their implementation, and gauge the extent to which the upcoming scientific information might enhance the decision-making process.

### Learn so you can teach

The process of incorporating scientific information into decision making is not a simple one and could frustrate the most seasoned decision maker. Some aspects of the process are particularly effective; others are not. How do we keep a record of these tried-and-tested practices? The vantage point that decision makers hold from their front-row seat gives them a unique opportunity to learn about the properties of the scientific supply of information they received and the intricacies of using this evidence to make informed decisions. One modest—but valuable—step is to iteratively document the strengths and limitations of the process regarding the discovery, assembly, and integration of scientific evidence to support each particular decision. What aspects of the scientific contribution (e.g., format, timing, discipline, dissemination approach) worked best? What of this contribution was most challenging to grasp and how could it be improved? Did the available scientific information appear to simply pursue academic requirements, or did it deliver tangible information so that end users could shape high-confidence decisions? (Hyman et al. 2022). The answer to these questions could help clarify how future priority decision contexts could benefit from scientific information and how to calibrate the needed level of engagement with scientists during early planning stages of proposed research. Learning from an assessment of the scientific process and its outputs offers multiple benefits. Most importantly, the assessment contributes to building an invaluable record that: (1) contains practical insights toward procuring actionable science for the next decision; (2) guards against past mistakes; (3) serves as a memory refresher for those currently responsible for making decisions; and (4) provides background reference to future generations of decision makers.

The opportunity to learn does not stop there, and curious decision makers explore deeper questions during the post-decision space. Instead of focusing on the scientific evidence that contributed to a decision, they concentrate on the outcomes that, following a decision, unmask the success or failure of achieving targets of interest (Gertler et al. 2016). Did the final decision—once implemented—work as intended to deliver the anticipated mid- to long-term outcomes? This question, or some version of it, is at the heart of impact monitoring and evaluation efforts abundantly described in the literature (e.g., Baylis et al. 2016; Gertler et al. 2016). A more nuanced examination introduces the concept of research impact evaluation (Reed et al. 2021) which assesses whether the contribution of a body of research to a final outcome was “necessary” or “sufficient.” In other words, how meaningful (or not) was the scientific complement to the formulation of the final decision and, consequently, the observed final impact? Assessing the quality of decisions made and their final effect could highlight key aspects to consider when seeking scientific information again for the formulation of future decisions. Despite the interest in documenting impact and learning from it, however, these considerations often encounter methodological challenges, funding shortages, political resistance, and require long-term commitments before results begin to reveal themselves (Gertler et al. 2016; Knight et al. 2019). Regardless, these examples illustrate some options available to decision makers wishing to become active agents of change. In their role, they are in a privileged position to forestall future aggravation and laments that they or other colleagues may experience when science and decisions are far apart from each other.

### Support the scientific enterprise

Participating actively in the scientific enterprise is a powerful antidote to counteract the decision-maker’s lament. The generation of actionable scientific knowledge is not spontaneous or done in isolation. To prosper and grow, it requires a dynamic environment where personal, institutional, social, political, and financial support are essential. The lifecycle of scientific undertakings is far from perfect. Products and services may be slow, expensive, and lead to more—rather than less—controversy.

There are many opportunities to contribute to the effective production of actionable science. In their capacity as competent authorities who are the target end users of scientific outputs, decision makers could contribute in any way possible to be part of the journey leading to the science they hope to find. If their agency is willing to finance the impending science, they may engage to secure that commitment. Or they may engage influential contacts within their networks that may help achieve and maintain the necessary conditions for the sustainability of research. Similarly, they can assume a leadership role to forge collaborative partnerships that nourish co-creation, communication, commitment, and continuous review (Zaman et al. 2020). Decision makers—or their official designees—may champion the science they wish they had by serving on review boards and advisory committees where they can participate in proposal review, prioritization, and selection to, most essentially, voice their unique perspectives concerning the science contributions they need. Their engagement will support critical relationships (with scientists and other science and policy leaders), and introduce ideas to help streamline the delivery, receipt, and interpretation of scientific findings. This engagement, when initiated early in the decision-making process and sustained through to the end (but see Bamzai-Dodson et al. 2021), can yield more positive outcomes. Perhaps one of the most important implications of decision makers embracing a genuine interest in facilitating the conditions that lead to science they can use goes beyond serving their own self-interest: their actions may help improve the public’s understanding of the impact of science in their everyday life as it informs regulatory decision-making and policies at every level (Pidgeon and Fischhoff 2011).

## Final thoughts

The considerations offered here may allow decision makers to engage in thoughtful inner contemplation to alleviate their frustration if, for various reasons, evidence-based decision making does not seem possible, and the final selection of a decision alternative must be adopted without the benefit of consulting scientific evidence. Each consideration leads to a much deeper elaboration and discussion, supported by contributions from multiple scholars. By necessity, and due to space limitations, their treatment here was inevitably superficial.

While these considerations are aimed at decision makers who lament not having the scientific evidence they need to inform their decisions, it is ironic that many of them also apply to researchers who lament the lack of support for the science they wish to deliver (Bisbal 2022). My perspective regarding this statement has evolved over three decades of professional engagement with research organizations, policy experts, and administrators focused on the management of terrestrial, freshwater, and marine ecosystems at regional, national (U.S.), and international scales. This experience has exposed me to the diverse beliefs, values, and norms held by those involved in knowledge-production, knowledge-transfer, and knowledge-assimilation processes. To both communities, the take-home message could be summarized into two main thoughts. The first one has to do with the simplest notion at the foundation of actionable science: *To be actionable, the production of science applicable to a pending decision requires knowing the context for that decision beforehand*. The lack of clearly articulated decision contexts is the fatal flaw of many would-be actionable scientific endeavors. Understanding this key concept is vital to decision makers and scientists alike. Can decision makers unequivocally confirm that they have comprehensively framed the priority decision context that occupies them? If not, the expectation of receiving scientific information they can use is fortuitous at best. Can scientists unequivocally confirm what priority decision context will benefit from the scientific knowledge they intend to deliver? If not, their scientific output may not be actionable at all.

The second thought emerging from this article addresses a familiar aspect of human psychology: the behavior of those who concentrate in fault-finding and the shifting of blame on to others as a way to explain their misfortunes (e.g., Lozano and Laurent 2019). Once again, both decision makers and scientists lamenting their woes assign blame at the other as if trading accusations for the imperfections and deficiencies that afflict them. To the decision maker, deficiencies in the scientific information they encounter imply shortcomings among science producers or the entire scientific enterprise. To the scientist, the lack of funding or support for their proposed studies, or the lack of uptake of their products, insinuate ineptitude among funders and science users. While blaming others may be a tempting impulse to explain hardships and unmet expectations, inner contemplation may be more productive in helping both parties reach their intended goals.

The process of inner contemplation as a path to thoughtful self-discovery and awareness could materialize in two ways: one is *reflection*, which involves the act of looking back at past experiences to extract meaning and learn for future actions (e.g., Criado-Perez et al. 2023). The other is *introspection* (or “reflexivity”, see Ryan 2007), which allows us to gain a critical viewpoint and awareness of how our personal values, emotions, understandings, and perspectives influence our perception of a current experience. Because the framing of decision contexts and the generation of knowledge to inform them often involve integrating a plurality of voices, numerous viewpoints, and cross-disciplinary epistemologies, both decision makers and scientists have ample opportunities to practice reflection and introspection to identify and negotiate their perspectives as part of an ongoing social process. The interwoven motivation of these two practices makes them perhaps inseparable. They both aim at reversing outward blame, bringing much needed humility to their professional practices. Through that mindful habit, decision makers and scientists will be able to find pragmatic answers to this question: What can I do within my own capacity to ensure that the necessary actionable science becomes available and facilitate its use to inform decisions? The answers are sure to inspire many and, undoubtedly, help alleviate the lament load.

## Data Availability

This article is a Perspective and does not contain data.
